# Safety and Effectiveness of Sodium-Glucose Co-Transporter 2 Inhibitors in Active Cancer Patients with Heart Failure: Results of the Observational TOSCA Trial

**DOI:** 10.3390/jcdd12090354

**Published:** 2025-09-13

**Authors:** Maria Laura Canale, Iacopo Fabiani, Maria Grazia Delle Donne, Michela Chianca, Valentina Barletta, Eugenia Capati, Monica Solinas, Lara Frediani, Elio Venturini, Giuseppe Arena, Giulio Zucchelli, Emilio Maria Pasanisi, Domenico Amoroso, Giacomo Allegrini, Raffaele De Caterina, Michele Emdin, Andrea Camerini

**Affiliations:** 1 Department of Cardiology, Versilia Hospital—Azienda USL Toscana Nord Ovest, 55041 Lido Di Camaiore, Italy; marialaura.canale@uslnordovest.toscana.it (M.L.C.); monica.solinas@uslnordovest.toscana.it (M.S.); 2 Department of Cardiology, Fondazione Toscana Gabriele Monasterio, 56124 Pisa, Italy; iacopofabiani@gmail.com (I.F.); emdin@ftgm.it (M.E.); 3 Department of Cardiology, UO Cardiologia 1, Azienda Ospedaliero Universitaria Pisana, 56122 Pisa, Italy; mgdd@virgilio.it (M.G.D.D.); raffaele.decaterina@unipi.it (R.D.C.); 4 Scuola di Specializzazione Cardiologia, University of Pisa, 56122 Pisa, Italy; michela.chianca@gmail.com; 5 Department of Cardiology, UO CARDIOLOGIA 2, Azienda Ospedaliero Universitaria Pisana, 56122 Pisa, Italy; v.barletta@ao-pisa.toscana.it (V.B.); g.zucchelli@ao-pisa.toscana.it (G.Z.); 6 Department of Cardiology, Livorno Hospital, Azienda USL Toscana Nord Ovest, 57100 Livorno, Italy; eugenia.capati@uslnordovest.toscana.it (E.C.); emiliomaria.pasanisi@uslnordovest.toscana.it (E.M.P.); 7 Department of Cardiology, Villamarina Hospital, Azienda USL Toscana Nord Ovest, 57025 Piombino, Italy; lara.frediani@uslnordovest.toscana.it; 8 Unit of Cardiac Rehabilitation, Azienda USL Toscana Nord Ovest, Cecina Hospital, 57023 Cecina, Italy; elio.venturini@uslnordovest.toscana.it; 9 Department of Cardiology, Nuovo Ospedale Apuano, Azienda USL Toscana Nord Ovest, 54100 Massa, Italy; giuseppe.arena@uslnordovest.toscana.it; 10 Department of Medical Oncology, Versilia Hospital, Azienda USL Toscana Nord Ovest, 55041 Lido Di Camaiore, Italy; domenico.amoroso@uslnordovest.toscana.it; 11 Department of Medical Oncology, Livorno Hospital, Azienda USL Toscana Nord Ovest, 57100 Livorno, Italy; giacomo.allegrini@uslnordovest.toscana.it

**Keywords:** SGLT2i, heart failure, safety, effectiveness, cancer, gliflozins

## Abstract

Cancer patients have not been included in landmark trials of SGLT2is in heart failure, so data on safety and effectiveness are lacking. TOSCA is a multi-center observational trial including patients with active cancer receiving SGLT2is for HF treatment. The primary endpoint was safety, and the secondary endpoint was effectiveness. Exploratory endpoints included drug–drug interactions, treatment of cancer therapy-related cardiac dysfunction (CTRCD), and changes in NT-proBNP. One-hundred and twenty-nine patients (median age 72 [range 44–92] yrs) were enrolled who had been receiving SGLT2i for a median of 3 (range 3–25) months. Prevalent etiology was drug-induced HF with HFrEF as the most frequent clinical presentation. The incidence of urinary tract infections was 1.8%, with no cases of genital infections, hypoglycemia, diabetic ketoacidosis, acute renal injury, thrombosis, or bone fractures. The mean overall EF increased (40.3% vs. 47.4%), and NYHA class improved in 19% of cases. Rates of unplanned cardiology visits (0.9%), use of i.v. diuretics (0.9%), coronary angiography (4.5%), emergency access for HF (1.8%), and new HF episodes (3.6%) were extremely low. In 11 cases (8.5%), the initiation of SGLT2i enabled continuation of anticancer therapy that would have otherwise been delayed or suspended due to HF decompensation. SGLT2is appeared effective in 34 cases of CTRCD. No drug–drug interactions were reported. SGLT2is confirmed their safety and effectiveness in active cancer patients with HF, with a potential cardioprotective effect. No new safety warnings were recorded.

## 1. Introduction

Sodium-glucose co-transporter 2 inhibitors (SGLT2is), also known as gliflozins, represent a novel class of oral agents originally developed for type 2 diabetes mellitus (T2DM). By inhibiting renal glucose and sodium reabsorption, these drugs exert glucose-lowering and natriuretic effects [1]. Beyond glycemic control, SGLT2is have demonstrated significant cardiovascular benefits [2]. The efficacy of SGLT2is in heart failure (HF) has been tested in different HF phenotypes, proving them beneficial in reducing both cardiovascular mortality and HF-related hospitalization incidence in patients with HF with reduced ejection fraction (HFrEF) (up to 30–35%) [3,4], as well as a reduction in HF-related hospitalizations in patients with HF with preserved ejection fraction (HFpEF) [5,6]. These benefits extend to patients with and without atherosclerotic cardiovascular disease or prior HF, positioning SGLT2is as a valuable option for HF prevention in at-risk populations [7]. Furthermore, SGLT2is are recommended for the prevention of HF in stage A patients at increased risk, underscoring their broader cardiovascular protective role [8,9].

Cancer therapy-related cardiac dysfunction (CTRCD) encompasses a spectrum of cardiovascular complications, including left ventricular dysfunction and HF, myocarditis, and arrhythmias [10]. These conditions present significant clinical challenges and negatively impact patient outcomes. Furthermore, consistent data indicate an elevated incidence of cardiovascular disease among cancer survivors, with HF development adversely affecting cancer prognosis [11]. Despite the growing recognition of CTRCD, effective cardioprotective strategies remain scarce, underscoring the urgent need for alternative therapeutic options. As cancer patients have been largely excluded from pivotal clinical trials, the impact on CTRCD of SGLT2is in ischemic and non-ischemic cardiomyopathy remains underexplored. Emerging evidence suggests potential cardioprotective effects of SGLT2is in oncologic patients with diabetes undergoing anthracycline-containing chemotherapy, showing how these agents may help mitigate CTRCD [12,13]. Large-scale clinical trials assessing the impact of SGLT2is on incident HF in broad oncologic populations remain a critical unmet need. Additionally, it is unclear whether SGLT2is may influence survival outcomes in patients undergoing cancer treatment. Moreover, no prospective safety and effectiveness data on active cancer patients are available for SGLT2is to date, making this topic of primary relevance in daily clinical practice. Addressing these knowledge gaps could significantly refine cardiovascular care strategies in oncology patients, reinforcing the expanding role of SGLT2is beyond diabetes management and HF treatment, while also highlighting the limited safety data in patients with active cancer. To fill these knowledge gaps, we conducted a multi-center study consisting of a retrospective part and a prospective part to assess the safety and impact on cardiovascular outcome of SGLT2is in patients with a broad spectrum of active tumors undergoing chemotherapy.

## 2. Materials and Methods

### 2.1. Subjects and Study Design

TOSCA is a multi-center observational retrospective and prospective non-profit study. Patients were recruited across 6 centers in Italy between 1 March 2022, and 1 March 2024, with a planned enrollment range from 80 to 200 participants; given the observational design of the study no formal sample size was defined. Patients enrolled in the prospective cohort underwent a three-month observation period (concluding on 1 June 2024), with two scheduled visits (baseline and follow-up). For patients in the retrospective cohort, data were obtained from a single index visit.

The TOSCA study enrolled adult patients (aged ≥18 years) with active cancer, defined as a histologically or cytologically confirmed solid or hematologically active malignancy or a history of neoplasm, and who had undergone radical treatment within the previous 3 years. Eligible patients must have had a history of HF, with either preserved or reduced ejection fraction, on treatment with primarily approved SGLT2i for HF (empagliflozin or dapagliflozin). All patients were required to be capable of providing informed consent. Recruitment comprised both outpatient and inpatient settings, including ambulatory and day hospitals/services. Patients with non-melanoma skin cancers, in situ breast cancer, or localized cervical cancer who had undergone radical treatment were excluded. These malignancies were excluded as they typically require less cardiotoxic treatment and have a limited impact on cardiovascular outcomes, potentially confounding the study’s objectives focused on active, systemic cancer therapies. The final study population included 129 patients (63 males and 66 females). Baseline comorbidities included hypertension (56%), coronary artery disease (30%), and diabetes mellitus (21%). No mortality was observed during follow-up.

Data collected included baseline demographics, cardiovascular risk factors, previous or concomitant heart disease, home therapy, the type of SGLT2i inhibitor used, tumor site, stage, cancer treatment, ongoing supportive care drug therapy (opioids, anti-emetics, anti-fungals), reported treatment-related toxicities (hypoglycemia [blood glucose ≤ 70 mg/dL], urinary tract infections, genital infections, hypovolemia, hypotension, acute renal failure, bone fractures, amputations, diabetic ketoacidosis, thromboembolic events, hypokalemia [K^+^ < 3.5 mEq/L or <3.5 mmol/L], and hyperkalemia [K^+^ > 5.5 mEq/L or >5.5 mmol/L]), response to treatment with empagliflozin or dapagliflozin (defined as changes in New York Heart Association—NYHA class, and cardiovascular events), and analysis of clinical and bio-humoral parameters.

The study protocol conforms to the ethical guidelines of the 1975 Declaration of Helsinki and received formal approval from the Institutional Ethical Review Board—Comitato Etico Area Vasta Nord Ovest (CEAVNO—approval number 23753/23 on 18 May 2023), and all patients provided consent for study enrollment and for the use of their anonymized data for research purposes in compliance with privacy regulations.

### 2.2. Follow-up and Endpoints

Patient follow-up was concluded on 1 June 2024. The primary endpoint was drug safety, evaluated by the incidence of both known and unexpected adverse events of glifozines (compared to safety data from registry studies: DAPA-HF [3], EMPEOR REDUCED [4], EMPEROR PRESERVED [5], and DELIVER [14]). The secondary endpoint was treatment efficacy assessed by improvements in ejection fraction (EF), changes in NYHA functional class, and HF-related cardiovascular events (including hospitalization and urgent/emergency visits for HF). As exploratory endpoints, the study aimed to evaluate the changes over time in cardiac biomarkers of cardiotoxicity (including N-terminal pro-B-type natriuretic peptide (NT-proBNP) and troponins), the potential clinically detectable drug interactions between gliflozines and cancer/supportive therapies, and the impact of SGLT2 in terms of CTRCD.

### 2.3. Statistical Analysis

Statistical analyses were conducted using R software (version 3.4.0) and SPSS (version 25.0, 2017, IBM Statistics, Armonk, NY, USA). A two-tailed *p*-value of ≤0.05 was considered statistically significant. Quantitative variables were expressed as mean ± standard deviation (SD) or median with interquartile range (IQR), depending on data distribution, while qualitative variables were reported as frequencies and percentages. Data analysis was conducted on both the general study population and separately on retrospective and prospective sub-groups. The chi-square test for categorical variables and Student’s t-test or the Mann–Whitney U-test for continuous data, respectively, were used to compare baseline characteristics. All patients meeting the inclusion criteria were enrolled consecutively to minimize selection bias. Due to the study’s observational nature, no additional pharmacological intervention was planned, and no intergroup comparison was intended.

## 3. Results

### 3.1. Study Population

Between 1 June 2023, and 1 March 2024, 129 patients were enrolled, 88 (68%) in the prospective cohort and 41 (32%) in the retrospective one, in six Italian centers. The median age of the general population was 72 (range 45–92) years with female patients accounting for 49% of cases. Regarding SGLT2i, patients received either dapagliflozin (58%) or empagliflozin (42%) on top of guideline-directed HF medical therapy for a median of 3 (range 3–25) months. The prevalent etiology was drug-induced HF (35%), with HFrEF as the most frequent clinical presentation (44%); we observed a trend toward an increase in the use of SGLT2is in patients with HFpEF. A total of 34 patients (26%) presented with clear features of cancer therapy-related cardiac dysfunction (CTRCD), as defined by previous exposure to cardiotoxic agents and echocardiographic evidence of left ventricular dysfunction. The main cancer sites were the breast, gastrointestinal tract, and hematological malignancies. Most cases (73%) received an active treatment mainly consisting of chemotherapy and target agents (mostly acalabrutinib, ibritinib, lapatinib, imatinib, capecitabine, osimertinib, and lenalidomide) while on SGLT2i. Palliative radiotherapy was delivered in only 17.8% of patients and always before SGLT2i treatment. Study population characteristics did not differ when considering retrospective and prospective cohorts separately. Study population characteristics are presented in Table 1 for the general, retrospective, and prospective cohorts.

### 3.2. Safety

Overall, SGLT2is were extremely safe and well tolerated in active cancer patients. Patients declared high compliance with gliflozin therapy. The incidence of urinary tract infections (one of the most frequent side effects reported in the general population) was low, with 1.6% of patients reporting this side effect. Since the majority of cases were on active cancer treatment with classic chemotherapy, with vomiting as one of the main toxicities, we focused on the rate of serum potassium disorders; hyperkalemia and hypokalemia were reported in 6.2% and 1.6% of cases, respectively. Interestingly no relation with the occurrence of vomiting was noted. No cases of genital infections, hypoglycemia, diabetic ketoacidosis, acute renal injury, thrombosis, amputations, or bone fractures were reported during the observation period. No patients discontinued gliflozin treatment due to side effects. Table 2 summarizes the safety items of SGLT2is in the TOSCA trial.

No new safety warnings or unexpected toxicities were reported. We found no clinically detectable drug–drug interactions with anticancer therapies and supportive care drugs (opioids, anti-emetics and anti-fungals). Similarly, no clinically detectable interaction with pre-existing cardiology therapy was noted. No liver toxicity or hepatic enzyme alterations were reported. Kidney function remained stable throughout follow-up, with no cases of acute kidney injury. No ischemic events (e.g., myocardial infarction, stroke) or arrhythmias were observed.

### 3.3. Effectiveness in General Population and in CTRCD

Active cancer patients could represent a challenging HF population due to the high rate of comorbidities, polypharmacy, concomitant side effects of anticancer treatment, and reduced performance status in advanced disease. So, the efficacy of cardiovascular drugs could not be fully appreciated. Contrary to these premises, SGLT2is confirmed to be effective in the study population, even taking into account the relatively short observation time. The mean overall ejection fraction in the whole population increased from 40.3 ± 9.6% to 47.4 ± 7.8%; data on separate cohorts showed a similar increase in both retrospective (from 36.2 ± 12.1% to 44.8 ± 5.5%) and prospective (from 43.6 ± 9.1% to 49.6 ± 8.3%) ones. A parallel reduction in mean NT-proBNP levels (2340 ± 5042 vs. 904 ± 1725 pg/mL, *p* = 0.055) was observed. Rates of unplanned cardiology visits (0.9%), use of i.v. diuretics (0.9%), coronary angiography (4.5%), emergency access for HF (1.8%), and new HF episodes (3.6%) were extremely low. Death events (cardiovascular and non-cardiovascular) were not reported.

NYHA class improved in 19% of cases (25 out of 129 patients), with 19 cases moving from NYHA III to II and 6 cases from NYHA II to I. Of note, the majority of the cases with NYHA class improvement belonged to the category of drug-induced HF, supporting the efficacy of gliflozins in cardiac toxicity. Focusing on cardiac toxicity patients, SGLT2is appeared effective in the 34 cases of clear CTRCD in the prospective cohort (clinical charts independently reviewed by two experts in cardioncology—MLC and IF); apart from the above-reported improvement in NYHA class, we observed a parallel improvement in both LVEF (40.1 ± 9.6 vs. 45.8 ± 9.7) and global longitudinal strain (GLS) (−15.1 SD 2.9% vs. −18.8 SD 2.1%). Moreover, a significant reduction in mean NT-proBNP levels (3855 ± 7391 vs. 649 ± 313 pg/mL; 95% [CI 166–6244], *p* = 0.039) was reported in this subgroup. In 11 cases (8.5%), the initiation of SGLT2i enabled continuation of anticancer therapy that would have otherwise been delayed or suspended due to HF decompensation. Taken together, all these data support the efficacy of gliflozins in complex cases of CTRCD.

## 4. Discussion

The findings of this multi-center observational study underscore the promising potential of SGLT2 inhibitors (dapagliflozin and empagliflozin) in active cancer patients with HF, including those with cancer therapy-related cardiac dysfunction (CTRCD). The significant increase in left ventricular ejection fraction (LVEF) and the notable reduction in NT-proBNP levels suggest a strong cardioprotective effect, even within a population characterized by multiple comorbidities, polypharmacy, and a high cardiovascular risk profile. Given that cancer patients have historically been excluded from major cardiovascular trials, our study provides valuable real-world evidence supporting the integration of SGLT2is into the cardio-oncology landscape. These results align with emerging data suggesting that SGLT2i may have a role beyond glycemic control and HF management, potentially exerting direct cardioprotective effects against chemotherapy-induced cardiac dysfunction [13]. Moreover, this study complements prior large-scale trials in non-oncologic HF populations, such as DAPA-HF and EMPEROR-Reduced, which demonstrated reductions in HF-related hospitalizations and cardiovascular mortality [3,4].

Notably, SGLT2is demonstrated an excellent safety profile in this cancer cohort, with minimal adverse events such as urinary tract infections (1.6%) and hyperkalemia (6.2%) and no reported cases of diabetic ketoacidosis, hypoglycemia, or thromboembolic events. This reassuring safety profile is particularly relevant given the high-risk nature of cancer patients, many of whom undergo intensive chemotherapy, immunotherapy, or targeted therapies that can exacerbate cardiovascular complications. While recent studies have raised concerns about the increased risk of acute kidney injury (AKI) and electrolyte imbalances in oncologic populations, our findings indicate that SGLT2is did not contribute to worsening renal function. A growing body of literature suggests that SGLT2is may provide nephroprotection in cancer patients, particularly those receiving nephrotoxic agents such as cisplatin or immune checkpoint inhibitors [15]. Furthermore, the nearly equal gender representation (51% male, 49% female) supports the generalizability of these findings across the sexes, reinforcing the potential applicability of SGLT2is in both male and female cancer patients. Additionally, the absence of significant drug–drug interactions with oncological treatments, including chemotherapy and targeted agents, underscores their compatibility with the complex treatment regimens of cancer patients.

The prospective data suggest a more significant improvement in HF outcomes than the retrospective cohort, likely due to better monitoring and adherence to treatment. The observed shift in NYHA class in 19% of cases, with patients transitioning from severe to milder forms of HF, reinforces the clinical benefit of SGLT2i in this high-risk population. Notably, chemotherapy-induced HF, a frequent and severe consequence of anthracyclines and other cardiotoxic cancer therapies, appeared to respond favorably to SGLT2i therapy. A recent meta-analysis examining the effects of SGLT2is in cancer patients with diabetes undergoing chemotherapy reported a significant reduction in HF-related hospitalizations and cardiovascular mortality [12]. These findings suggest that SGLT2is could be a viable cardioprotective strategy in oncology patients at risk of developing CTRCD. Mechanistically, preclinical models have demonstrated that SGLT2is reduce oxidative stress, myocardial fibrosis, and inflammation, key contributors to chemotherapy-induced cardiotoxicity. This reduction in oxidative stress, fibrosis, and inflammation could be a potential mechanism for the observed cardioprotective effects of SGLT2is [16].

All these findings strengthen previous evidences from the studies in [15,17,18].

However, several questions remain regarding the long-term impact of SGLT2is in oncology patients. Whether their cardiovascular benefits translate into improved overall survival or reduced cancer progression is still unclear. This underscores the need for further research and engagement in the ongoing development of cardio-oncology. Furthermore, the study raises important questions regarding whether the cancer stage influences the efficacy of SGLT2is, as both early and advanced malignancies were included. While our study included both early and advanced malignancies, future subgroup analyses are necessary to determine whether tumor burden, systemic inflammation, or cachexia influence the cardiovascular benefits of these agents in oncology patients. Additionally, more extended follow-up studies are needed to confirm these preliminary findings and assess whether SGLT2is may impact long-term cardiovascular and oncologic outcomes in this unique patient population.

Despite its valuable insights, the study has several limitations. The short follow-up period in the prospective cohort (three months) restricts the ability to draw conclusions on the long-term safety and effectiveness of SGLT2is in this population. More extended observation periods are necessary to assess sustained benefits and delayed adverse effects. Additionally, the retrospective part introduces potential reporting bias, as clinical documentation may not have captured all relevant patient data or treatment outcomes. This could impact the accuracy of adverse event reporting and response assessments, necessitating cautious interpretation of retrospective findings. Additionally, the relatively small sample size (n = 129) may limit generalizability, especially when compared to larger registry-based studies.

The lack of a control group limits the ability to directly compare SGLT2i outcomes against standard HF therapy alone, making it challenging to definitively attribute improvements solely to these agents. Furthermore, while the multi-center design strengthens the study’s external validity, the relatively small sample size may not fully capture the heterogeneity of cancer patients with HF. Variability in cancer types, treatment regimens, and patient characteristics could influence responses to SGLT2is, necessitating larger, randomized controlled trials to confirm these findings. Lastly, while improvements in cardiovascular markers are encouraging, this study does not address whether these translate into improved overall survival or better oncological outcomes.

## 5. Conclusions

This study provides compelling evidence that SGLT2 inhibitors are both safe and effective in managing heart failure in cancer patients, including those affected by cancer therapy-related cardiac dysfunction. The improvements in ejection fraction, NT-proBNP levels, and NYHA class indicate a potential cardioprotective role for these drugs beyond their conventional use in diabetes and HF management. Importantly, their excellent safety profile, absence of significant drug–drug interactions, and similar benefits across both male and female patients reinforce their potential as an adjunctive therapy in oncologic cardiology, offering a promising new approach to managing heart failure in cancer patients.

However, the short follow-up duration and the retrospective nature of part of the study pose limitations that require further investigation. The lack of a control group and relatively small sample size highlight the urgent need for larger, prospective, randomized trials to confirm these preliminary findings. Future research should focus on long-term cardiovascular and oncological outcomes to fully elucidate the role of SGLT2is in cancer patients with heart failure.

## Figures and Tables

**Table 1:** Study population characteristics for overall (n = 129), retrospective (n = 41), and prospective (n = 88) cohorts.

	Overall	Retrospective	Prospective
Median age [range]	72 [45–92] years	71 [45–92] years	73 [47–92] years
Male–Female	51–49%	55–45%	49–51%
Dapagliflozin Empagliflozin	58% 42%	61% 39%	56% 44%
Median treatment duration [range]	3 [3–25] months	6 [4–25] months	3 [3–3] months °
Clinical phenotype HFrEF HFmrEF HFpEF	44% 25% 31%	52% 25% 23%	41% 19% 40%
Etiology			
Drug-induced	35%	28%	39%
CAD	30%	32%	26%
CMPs	15%	13%	17%
Valve disease	12%	13%	11%
Hypertension	8%	14%	7%
Active treatment	73%	70%	75%
Cancer treatment			
Chemotherapy	53%	57%	51%
Target agents	28%	25%	30%
Immunotherapy	10%	8%	13%
Endocrine therapy	9%	10%	6%
Supportive treatment	61%	58%	63%
Cancer site			
Breast	28%	32%	26%
Hematological	26%	28%	25%
Gastro-intestinal	20%	21%	20%
Other *	26%	19%	29%
Cancer stage Early Advanced	68% 32%	71% 39%	66% 34%

HFrEF: heart failure with reduced ejection fraction; HFmrEF: heart failure with mildly reduced ejection fraction; HFpEF: heart failure with preserved ejection fraction; CAD: coronary artery disease; CMPs: cardiomyopathies. ° observation was set for 3 months in prospective cohort per protocol; * includes lung, head and neck, prostate, bladder, and gynecological cancer.

**Table 2:** Safety of SGLT2is in active cancer patients in overall (n = 129), retrospective (n = 41), and prospective (n = 88) cohorts.

	Overall	Retrospective	Prospective
Urinary tract infections	1.6%	4.8%	0%
Genital tract infections	0%	0%	0%
Hypokalemia	1.6%	0%	2.2%
Hyperkalemia	6.2%	12.1%	3.4%
Hypoglycemia	0%	0%	0%
Diabetic keto-acidosis	0%	0%	0%
Hypovolemia	0%	0%	0%
Symptomatic hypotension	0%	0%	0%
Acute renal failure	0%	0%	0%
Bone fractures	0%	0%	0%
Amputation	0%	0%	0%
Thromboembolic events	0%	0%	0%

## Data Availability

Upon reasonable request.

## Abbreviations

The following abbreviations are used in this manuscript:

AKI	acute kidney injury
CTRCD	cancer therapy-related cardiac dysfunction
EF	ejection fraction
HF	heart failure
HFpEF	heart failure with preserved ejection fraction
HFrEF	heart failure with reduced ejection fraction
IQR	median with interquartile range
LVEF	left ventricular ejection fraction
NT-proBNP	N-terminal pro-B-type natriuretic peptide
NYHA	New York Heart Association
SD	standard deviation
SGLT2i	sodium-glucose co-transporter 2 inhibitor
T2DM	type 2 diabetes mellitus
